# Application of tandem mass spectrometry for blood acylcarnitine and amino acid profiling in differentiating etiologies of neonatal cholestasis

**DOI:** 10.3389/fped.2026.1862155

**Published:** 2026-05-29

**Authors:** Haoxuan Sun, Ya Wang, Haoran Wu, Haoxuan Li, Shuhan Peng, Dong Zhang

**Affiliations:** 1 College of Traditional Chinese Medicine, Shandong University of Traditional Chinese Medicine, Jinan, Shandong Province, China; 2 Medical School, Shandong University of Traditional Chinese Medicine, Jinan, Shandong Province, China; 3 School of Nursing, Shandong University of Traditional Chinese Medicine, Jinan, Shandong Province, China; 4 Office of Laboratory Management, Shandong University of Traditional Chinese Medicine, Jinan, Shandong Province, China

**Keywords:** acylcarnitine, amino acid profiling, biliary atresia, diagnosis, differential diagnosis, neonatal cholestasis, tandem mass spectrometry

## Abstract

**Objective:**

This study aims to evaluate the clinical value of combined blood acylcarnitine (BA) and amino acid profiling in differentiating the etiologies of neonatal cholestasis (NC) and to provide a novel approach for the early and accurate identification of biliary atresia and non-biliary atresia forms of NC.

**Methods:**

Clinical data were retrospectively collected from 180 newborns with NC who were admitted to this hospital between January 2023 and January 2026. The patients were divided into a biliary atresia group and a non-biliary atresia group based on whether they ultimately developed BA. Baseline characteristics and laboratory test results were obtained for all participants. Plasma acylcarnitine levels were detected by tandem mass spectrometry, and serum amino acid concentrations were determined using high-performance liquid chromatography/tandem mass spectrometry. Acylcarnitine and amino acid concentrations were then compared between the two groups. The diagnostic ability of individual metabolites was assessed by calculating the area under the curve (AUC). A composite detection model was established by performing binary classification analysis, and its predictive accuracy was evaluated.

**Results:**

Compared with the non-biliary atresia group, patients in the biliary atresia group showed significantly higher levels of direct bilirubin/total bilirubin ratio, *γ*-glutamyl transpeptidase, alkaline phosphatase, C2, C8, C12, C18, leucine (Leu), valine (Val), proline, phenylalanine, alanine (Ala), glycine, and the (Leu + Val)/tyrosine ratio (all *P*’s < 0.05). Receiver operating characteristic curves of the individual markers showed that Leu had the highest discrimination index (AUC = 0.805), whereas C2 and Ala were less effective. The integrated detection system obtained an AUC of 0.963, with sensitivity exceeding 89.86% and specificity exceeding 86.24%.

**Conclusion:**

Overall, the combined evaluation of plasma acylcarnitine and amino acid profiles provides a more accurate approach for identifying the causes of neonatal cholestasis, such as biliary atresia, through multiple factors. This method provides necessary non-invasive reference for diagnosing neonatal cholestasis and should be widely promoted for clinical use.

## 1 Introduction

Neonatal cholestasis (NC) is a clinical syndrome characterized by impaired bile excretion due to structural or functional abnormalities of the intrahepatic or extrahepatic bile ducts, primarily presenting with elevated direct bilirubin (DBIL). Its incidence is approximately 1‰–3‰, making it one of the more common severe liver diseases during the neonatal period. If not diagnosed and treated promptly, it can progress to hepatic fibrosis, cirrhosis, or even liver failure, posing a serious threat to the life and health of affected infants (1, 2). However, the clinical manifestations of the disease are diverse and lack specificity, while its etiology is complex and multifactorial, including conditions such as biliary atresia, genetic metabolic disorders, and infections. Early identification of the cause of neonatal cholestasis remains a significant challenge, with clinical misdiagnosis rates exceeding 30%, thus delaying the optimal timing for precise intervention (3).

Regarding the epidemiology and clinical harm in the Chinese population, the incidence of biliary atresia shows significant ethnic and regional differences, with much higher rates in Asia than in Europe and America. According to a domestic multicenter study, the incidence of BA in Shanghai is approximately 10.86 per 100,000 live births (about 1 in 9,200), and infants who do not undergo timely Kasai surgery usually cannot survive with their native liver beyond 1 year of age (4, 5). In terms of etiological composition, the etiology spectrum of infantile cholestasis in China is dominated by biliary tract developmental abnormalities, with BA accounting for up to 49.4% of cases. Inherited metabolic liver diseases account for approximately 9.8%–20%, among which neonatal intrahepatic cholestasis caused by citrin deficiency (NICCD) is one of the most common types (6). These data indicate that the disease burden of neonatal cholestasis in the Chinese population is substantial and that its etiological composition differs somewhat from that observed in Western countries, making early and accurate differentiation particularly important.

Currently, etiological categorization of NC involves a combination of clinical presentation, biochemical tests, and imaging results. Ultrasound serves as the main imaging method and plays an indispensable role in observing the biliary system. However, differences in operator experience and other factors often limit its ability to distinguish between various causes of intrahepatic cholestasis clearly (7, 8). Conventional serological indicators, such as serum bilirubin, *γ*-glutamyl transpeptidase (GGT), and alkaline phosphatase (ALP), can indicate the presence of cholestasis but lack sufficient sensitivity and specificity for etiological differentiation, especially during the early stages of disease (9). Over the past several years, advances in genetic and metabolic detection technologies have revealed that some cases of neonatal cholestasis are closely related to amino acid and fatty acid oxidation disorders; therefore, metabolomic analysis can help determine their causes (10, 11).

Tandem mass spectrometry (MS/MS) can be used to identify many amino acids and acylcarnitines from a single dried blood spot without loss of information. It is frequently in neonatal screening for inherited metabolic diseases. Acylcarnitines are intermediate products of mitochondrial β-oxidation of long-chain fatty acids, and a significant increase suggests impaired mitochondrial fatty acid β-oxidation; conversely, alterations in amino acid patterns suggest disturbances such as urea cycle disorders and abnormalities in branched-chain amino acid metabolism. Research has shown that newborns with neonatal intrahepatic cholestasis caused by NICCD exhibit notable rises in serum citrulline (Cit), methionine, and several acylcarnitines (10, 12, 13). Wang et al. (10), through a retrospective evaluation of the missed NICCD instances, found that a combined analysis of Cit, glycine (Gly), phenylalanine (Phe), ornithine (Orn), and octanoylcarnitine (C8) increased the area under the receiver operating characteristic (AUC) curve from 0.953 to 0.996 compared with single-marker detection, indicating that multiple metabolic signatures are more effective in improving predictive accuracy. Gürcan Kaya et al. (14) reported that, in newborns with cholestasis, the combined analysis of GGT levels and metabolomics can more precisely differentiate among various pathological causes.

Currently, although many studies have focused on the use of blood acylcarnitine and amino acid profiling for etiological identification in newborns with NC, most have focused on specific causes. There remains a lack of systematic evaluation of the overall diagnostic effect of this approach across various causes and its superiority over single-metabolite analysis in clinical diagnosis. In addition, most research has been conducted in the West and lacks relevant data on Chinese children. Thus, to assess whether MS/MS-based blood acylcarnitine and amino acid profiling can reveal clinically useful clues regarding the source of NC. Furthermore, the study evaluates whether a combined detection approach can improve the sensitivity and specificity of an etiology-based recognition to boost the accuracy of identifying infants with NC through precise detection and correct treatment.

## 2 Materials and methods

### 2.1 General data

A retrospective was conducted on children diagnosed with NC at our hospital from January 2023 to January 2026. Based on the final diagnosis at discharge and illness course, the patients were divided into a biliary atresia group, comprising 76 individuals, and a non-biliary atresia group. This retrospective study was approved by the Institutional Review Board of Shandong University of Traditional Chinese Medicine. Due to the retrospective nature of the study and the use of anonymized data from existing medical records, the requirement for informed consent was waived by the ethics committee. The ethics committee does not issue approval numbers for this type of retrospective study. All data were anonymized prior to analysis.

The diagnosis of biliary atresia was confirmed by intraoperative biliary exploration and histopathological examination (5). Laparoscopic or open biliary exploration is currently regarded as the “gold standard” for the clinical diagnosis of biliary atresia. All patients included in the biliary atresia group were confirmed to have extrahepatic bile duct occlusion by intraoperative cholangiography. In addition, liver tissue samples were obtained during the operation for pathological examination, and the pathological features included typical features of biliary atresia, such as disruption of hepatic lobular architecture, marked proliferation of intrahepatic small bile ducts, and fibrous tissue proliferation in the portal area. Patients in the non-biliary atresia group were confirmed to have patent bile ducts by intraoperative cholangiography or had biliary atresia excluded by liver biopsy, and these patients were ultimately diagnosed with other forms of neonatal cholestasis based on clinical, biochemical, imaging, and pathological findings.

Inclusion criteria:

1. Diagnosis of neonatal cholestasis based on serum DBIL >17.10 μmol/L or >20% of total bilirubin (TBIL), with jaundice lasting more than 2 weeks.
2. Age between 7 days and 3 months after birth.
3. No prior surgical treatment or specific medication interventions for cholestasis.
4. Availability of comprehensive patient data and MS/MS metabolomic analysis outcomes.

Exclusion criteria:

1. Secondary cholestasis caused by external factors such as infection, parenteral nutrition-associated liver disease, or congenital heart disease.
2. Presence of other severe congenital diseases (e.g., severe congenital heart disease, sepsis).
3. Family history of liver disease.
4. Incomplete blood acylcarnitine and amino acid spectrum information.

### 2.2 Study methods

#### 2.2.1 Clinical data collection

Data were collected from the electronic medical record system, including the following:

1. Basic information: gender, postnatal age (days), and body weight.
2. Biochemical indicators: DBIL, TBIL, DBIL/TBIL, GGT, ALP, total bile acid (TBA), aspartate aminotransferase (AST), alanine aminotransferase (ALT), AST/ALT ratio, cholinesterase (CHE), immunoglobulins (Ig), glucose (Glu), blood ammonia (AMM), and alpha-fetoprotein (AFP).
3. Routine blood test indices: white blood cell count (WBC), platelet count (PLT), and hemoglobin (Hb).

#### 2.2.2 Blood acylcarnitine detection

Blood acylcarnitine concentrations were determined using MS/MS. Briefly, 10 µL of whole-blood sample was mixed with 900 µL of a chloroform:methanol (2:1 v/v) solution, along with an internal standard containing d3-propionylcarnitine. After vigorous stirring, the sample was centrifuged rapidly at 12,000 r/min for 10 min. The upper supernatant was collected afterward. The acylcarnitines identified included acetylcarnitine (C2), propionylcarnitine (C3), butyrylcarnitine (C4), valerylcarnitine (C5), hexanoylcarnitine (C6), octanoylcarnitine (C8), decanoylcarnitine (C10), lauroylcarnitine (C12), myristoylcarnitine (C14), palmitoylcarnitine (C16), and stearoylcarnitine (C18). The mass spectrometry conditions were as follows: ESI+ for electrospray ionization, source temperature set at 400 °C, nebulizer flow rate fixed at 10 L/min, collision gas pressure maintained at 10 psi, and a scanning range from 50 to 1,000 *m*/*z*.

#### 2.2.3 Amino acid spectrum detection

Serum amino acid concentrations were measured by high-performance liquid chromatography coupled with tandem mass spectrometry (HPLC–MS/MS). Briefly, 10 µL of serum was added to 90 µL of a 50% aqueous solution containing stable-isotope-labeled internal standards. After deproteinization and acid hydrolysis, the samples were separated by chromatography and subsequently analyzed by MS/MS. The amino acids analyzed included leucine (Leu), valine (Val), Cit, Orn, proline (Pro), arginine (Arg), methionine (Met), Phe, tyrosine (Tyr), alanine (Ala), and Gly. MS settings are as follows: ESI+, source temperature of 350 °C, nebulizer gas flow of 10 L/min, collision pressure of 10 psi, and a scanning range of 50–1,000 *m*/*z*.

### 2.3 Sample size justification

This study is a single-center retrospective diagnostic study aimed at developing a binary logistic regression model based on blood acylcarnitine and amino acid profiles to differentiate biliary atresia from non-biliary atresia etiologies in infants with neonatal cholestasis. According to the events-per-variable (EPV) rule of thumb for multivariable prediction models, logistic regression analysis generally requires a minimum of 10 positive events for each independent variable (15). In this study, univariate receiver operating characteristic (ROC) analysis was first performed to identify indicators with good discriminatory performance (*P* < 0.05 and AUC > 0.7), yielding eight candidate variables [C2, C12, Leu, Val, (Leu + Val)/Tyr, Pro, Ala, and Gly]. Based on the EPV ≥10 criterion, at least 80 positive events (i.e., biliary atresia cases) were required. Based on preliminary clinical data from our hospital and published literature, biliary atresia accounts for approximately 42%–48% of neonatal cholestasis cases (14). To ensure adequate model-fitting accuracy while maintaining sample representativeness, a total of 180 participants were finally enrolled, including 76 patients in the biliary atresia group. The resulting EPV was 9.5, which falls within the acceptable range and provides sufficient statistical power for the development of the multivariable diagnostic model.

### 2.4 Statistical analysis

Statistical analyses were performed using SPSS version 27.0. Continuous parameters were checked for normality. Data exhibiting normality are presented as mean ± standard deviation ( *x¯* ± *s*), and comparisons across groups were performed using the independent-samples *t*-test. For data that did not follow a normal distribution, the median with interquartile range was used for presentation, and the Mann-Whitney U test was employed for between-group comparisons. Categorical data are expressed as counts and percentages [*n* (%)] and were analyzed using the *χ*^2^ test or Fisher's exact test, as appropriate. ROC curve analysis was applied to evaluate the diagnostic accuracy of single- and multivariable metabolic indicators for distinguishing among different causes of neonatal cholestasis. A binary logistic regression framework was developed for integrated diagnostic analysis, and the AUC, sensitivity, and specificity were determined. *P* < 0.05 was considered statistically significant.

## 3 Results

### 3.1 Comparison of clinical data between the two groups

A total of 180 newborn infants diagnosed with cholestasis were enrolled, including 76 patients with confirmed biliary atresia and 104 patients without biliary atresia. No statistically significant differences were found between the two groups in terms of gender, postnatal age, and body weight, indicating that the patients are comparable.

In terms of biochemical indicators, the levels of DBIL/TBIL, GGT, and ALP were significantly higher in the biliary atresia group than in the non-biliary atresia group (*P* < 0.05). Among these markers, GGT showed the most significant difference between the two groups (528.63 ± 332.41 vs. 183.54 ± 98.72 U/L; *P* < 0.001). In addition, the Glu level in the biliary atresia group was lower than in the non-biliary atresia group (1.87 ± 0.76 vs. 2.42 ± 0.80 mmol/L; *P* = 0.014), suggesting that infants with biliary atresia are more susceptible to hypoglycemia. No substantial differences were observed between the cohorts with respect to TBIL, TBA, transaminases (AST, ALT), AST/ALT ratio, CHE, Ig, AMM, AFP, or standard blood parameters (WBC, PLT, Hb) (*P* > 0.05). Detailed results are presented in Table 1.

**Table 1:** Comparison of clinical data between the two groups.

Variable	Biliary atresia group (*n* = 76)	Non-biliary atresia group (*n* = 104)	*t*/*χ*^2^	*P*
Postnatal age (days)	45.53 ± 15.42	47.61 ± 14.21	0.102	0.919
Gender (male/female)	42/34	58/46	0.013	0.909
Weight (kg)	5.35 ± 0.76	5.42 ± 0.81	0.584	0.560
DBIL (μmol/L)	106.43 ± 38.09	102.45 ± 43.43	0.424	0.673
TBIL (μmol/L)	141.23 ± 49.11	139.66 ± 50.25	0.607	0.545
DBIL/TBIL	0.81 ± 0.09	0.75 ± 0.11	3.038	0.003
GGT (U/L)	528.63 ± 332.41	183.54 ± 98.72	7.287	<0.001
ALP (U/L)	712.21 ± 245.76	558.87 ± 173.64	3.905	<0.001
TBA (μmol/L)	138.78 ± 21.67	141.16 ± 20.35	0.721	0.472
AST (U/L)	69.43 ± 11.54	64.87 ± 10.77	0.383	0.703
ALT (U/L)	24.58 ± 5.76	27.01 ± 5.92	1.712	0.090
AST/ALT	3.42 ± 0.91	3.38 ± 0.88	0.294	0.769
CHE (U/L)	154.67 ± 55.78	161.07 ± 52.29	0.401	0.689
Ig (g/L)	15.21 ± 7.81	15.26 ± 8.03	0.591	0.556
Glu (mmol/L)	1.87 ± 0.76	2.42 ± 0.80	2.486	0.014
AMM (μmol/L)	85.78 ± 18.65	88.56 ± 20.12	0.658	0.512
AFP (ng/mL)	96,694.43 ± 36,953.11	96,435.65 ± 36,944.25	0.593	0.554
WBC count (×10^9^/L)	11.96 ± 4.15	11.88 ± 4.26	0.599	0.550
PLT level (×10^6^/L)	321.65 ± 118.43	332.54 ± 120.07	0.988	0.325
Hb content (g/L)	97.55 ± 17.21	101.56 ± 19.04	0.129	0.897

DBIL, direct bilirubin; TBIL, total bilirubin; DBIL/TBIL, ratio of directly bound to indirectly bound bilirubins in plasma; GGT, gamma-glutamyl transferase; ALP, alkaline phosphatase; TBA, total bile acids; AST, aspartate transaminase, ALT, alanine transaminase; AST/ALT, ratio between these two enzymes’ concentrations; CHE, cholinesterase activity; Ig, immunoglobulin content; Glu, blood glucose concentration; AMM, blood ammonia levels; AFP, alpha-fetoprotein; WBC, white blood cell; PLT, platelet; Hb, hemoglobin.

### 3.2 Comparison of blood acylcarnitines between the two groups

The blood acylcarnitine profile showed that the concentrations of C2, C8, C12, and C18 were significantly higher in patients with biliary atresia than in those without biliary atresia (*P* < 0.05). C2 showed the most significant difference between the two groups (27.54 ± 8.06 vs. 21.01 ± 9.23 μmol/L, *P* = 0.011). There were no statistically significant differences between the two groups in terms of the concentrations of C3, C4, C5, C6, C10, C14, and C16 (*P* > 0.05, Table 2).

**Table 2:** Comparison of blood acylcarnitines between the two groups (mean ± SD).

Variable	Biliary atresia group (*n* = 76)	Non-biliary atresia group (*n* = 104)	*t*	*P*
C2 (μmol/L)	27.54 ± 8.06	21.01 ± 9.23	3.531	0.011
C3 (μmol/L)	2.95 ± 0.98	2.85 ± 0.95	0.211	0.833
C4 (μmol/L)	0.26 ± 0.09	0.25 ± 0.11	0.214	0.831
C5 (μmol/L)	0.17 ± 0.09	0.18 ± 0.10	1.837	0.069
C6 (μmol/L)	0.06 ± 0.04	0.05 ± 0.02	1.479	0.142
C8 (μmol/L)	0.07 ± 0.04	0.05 ± 0.02	3.613	<0.001
C10 (μmol/L)	0.06 ± 0.03	0.06 ± 0.03	0.000	1.000
C12 (μmol/L)	0.10 ± 0.05	0.07 ± 0.04	4.679	<0.001
C14 (μmol/L)	0.26 ± 0.10	0.28 ± 0.16	1.308	0.193
C16 (μmol/L)	2.58 ± 1.12	2.51 ± 1.10	0.616	0.539
C18 (μmol/L)	0.73 ± 0.39	0.54 ± 0.24	3.333	0.001

C2, acetylcarnitine; C3, propionylcarnitine; C4, butyrylcarnitine; C5, valerylcarnitine; C6, hexanoylcarnitine; C8, octanoylcarnitine; C10, decanoylcarnitine; C12, lauroylcarnitine; C14, myristoylcarnitine; C16, palmitoylcarnitine; C18, stearoylcarnitine.

### 3.3 Comparison of serum amino acid levels

The serum amino acid profile showed that the levels of Leu, Val, Pro, Phe, Ala, and Gly and the (Leu + Val)/Tyr ratio were significantly higher in the biliary atresia group than in the non-biliary atresia group (*P* < 0.05). No substantial differences were observed between these cohorts in the concentrations of Cit, Orn, Arg, Met, or Tyr (*P* > 0.05, Table 3).

**Table 3:** Comparison of amino acid levels between the two groups (mean ± SD).

Variable	Biliary atresia group (*n* = 76)	Non-biliary atresia group (*n* = 104)	*t*	*P*
Leu (μmol/L)	112.32 ± 28.11	88.45 ± 22.31	3.864	<0.001
Val (μmol/L)	116.67 ± 27.25	90.04 ± 32.27	4.370	<0.001
(Leu + Val)/Tyr	3.52 ± 0.81	2.71 ± 0.81	3.937	<0.001
Cit (μmol/L)	25.39 ± 8.12	27.12 ± 10.53	1.330	0.186
Orn (μmol/L)	138.58 ± 34.43	127.32 ± 48.22	0.041	0.967
Pro (μmol/L)	162.34 ± 58.09	115.83 ± 44.19	4.572	<0.001
Arg (μmol/L)	32.45 ± 15.50	33.80 ± 16.04	1.749	0.083
Met (μmol/L)	28.33 ± 8.43	30.14 ± 10.13	1.325	0.118
Phe (μmol/L)	43.23 ± 10.11	35.16 ± 12.25	2.742	0.007
Tyr (μmol/L)	68.15 ± 16.24	72.26 ± 32.21	0.883	0.379
Ala (μmol/L)	262.24 ± 48.15	202.45 ± 86.17	4.245	<0.001
Gly (μmol/L)	332.45 ± 93.32	271.12 ± 106.45	2.516	0.013

Leu, leucine; Val, valine; Cit, citrulline; Orn, ornithine; Pro, proline; Arg, arginine; Met, methionine; Phe, phenylalanine; Tyr, tyrosine; Ala, alanine; Gly, glycine.

### 3.4 ROC curve analysis

#### 3.4.1 Diagnostic value of blood acylcarnitines in differentiating the etiology of neonatal cholestasis

**Figure 1:**
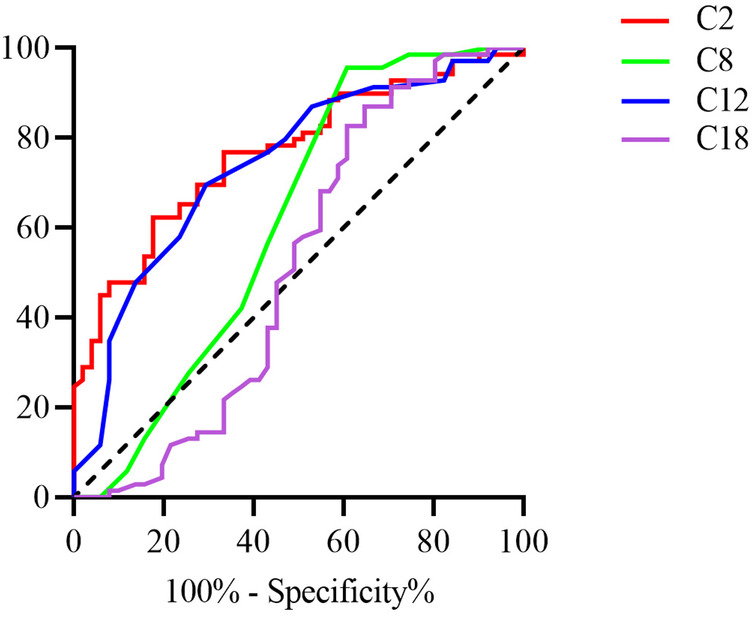
ROC curve for identifying the etiology of neonatal cholestasis utilizing acylcarnitine profiling.

ROC curves were generated to evaluate the discriminatory ability of serum acylcarnitines in distinguishing biliary atresia from non-biliary cholestasis. The AUC values for C2 and C12 were 0.767 (95% CI: 0.684–0.851) and 0.742 (95% CI: 0.652–0.832), respectively, both of which were statistically significant (*P* < 0.001).These findings suggest that C2 and C12 may be moderately helpful in determining the primary condition of infantile cholestasis. For C2, the Youden index was 0.447, with an optimal cutoff point yielding a sensitivity of 62.32% and a specificity rate of 82.35%. For C2, the Youden index was 0.402, with a sensitivity of 69.57% and a specificity of 70.59%. C8 showed restricted diagnostic ability, with an AUC of 0.615 (95% CI: 0.504–0.725, *P* = 0.032). In contrast, C18 showed no diagnostic value, with an AUC of 0.516 (95% CI: 0.401–0.630, *P* = 0.762). Detailed results are presented in Table 4 and Figure 1.

**Table 4:** Diagnostic value of blood acylcarnitines in differentiating the etiology of neonatal cholestasis.

Parameter	AUC	SE	*P*	95% CI	Youden index	Sensitivity (%)	Specificity (%)
C2	0.767	0.043	<0.001	0.684–0.851	0.447	62.32	82.35
C8	0.615	0.056	0.032	0.504–0.725	0.349	95.65	39.22
C12	0.742	0.046	<0.001	0.652–0.832	0.402	69.57	70.59
C18	0.516	0.059	0.762	0.401–0.630	0.223	86.96	35.29

#### 3.4.2 Value of amino acid spectrum analysis in differentiating the etiology of neonatal cholestasis

**Figure 2:**
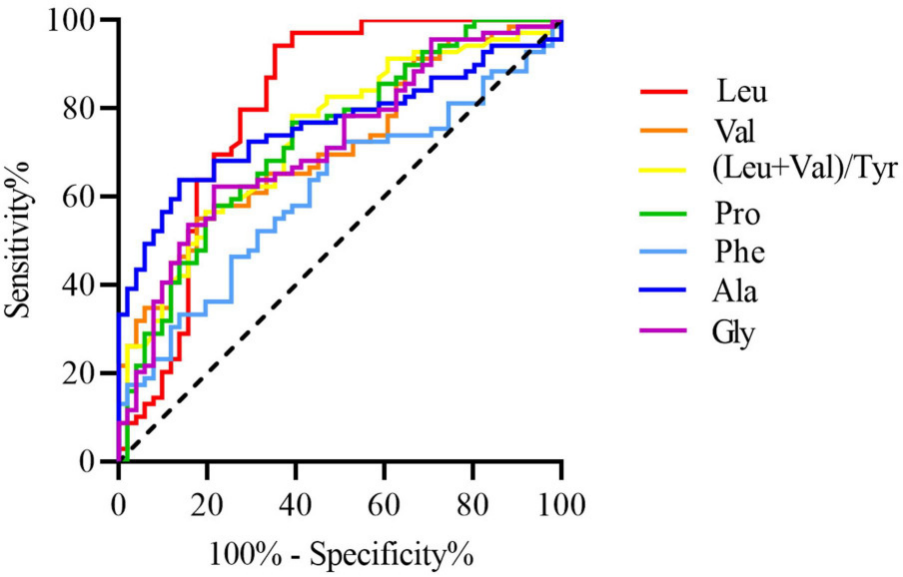
ROC curve for identifying various causes of neonatal jaundice through amino acid profile evaluation.

ROC curves were used to evaluate the discriminatory power of plasma amino acid levels in distinguishing biliary atresia from non-biliary atresia cholestasis. Leu demonstrated high diagnostic value, with an AUC of 0.805 (95% CI: 0.716–0.893, *P* < 0.001). At the optimal cutoff value, the Youden index was 0.589, corresponding to a sensitivity of 94.20% and a specificity of 64.71%. Ala showed good diagnostic performance, with an AUC of 0.760 (95% CI: 0.674–0.846, *P* < 0.001). The Youden index was 0.500, with a sensitivity of 63.77% and a specificity of 86.27%. The (Leu + Val)/Tyr ratio yielded an AUC of 0.735 (95% CI: 0.646–0.824, *P* < 0.001), while Pro, Val, and Gly demonstrated AUC values of 0.730 (95% CI: 0.639–0.820, *P* < 0.001), 0.713 (95% CI: 0.623–0.804, *P* < 0.001), and 0.717 (95% CI: 0.625–0.808, *P* < 0.001), respectively. Together, these parameters demonstrated moderate diagnostic value for distinguishing among different underlying causes of neonatal cholestasis. Phe did not show strong predictive power and exhibited an AUC of 0.612 (95% CI: 0.512–0.713, *P* = 0.036). Detailed results are presented in Table 5 and Figure 2.

**Table 5:** Diagnostic value of amino acid profiling in differentiating the etiology of neonatal cholestasis.

Variable	AUC	SE	*P*	95% CI	Youden index	Sensitivity (%)	Specificity (%)
Leu	0.805	0.045	<0.001	0.716–0.893	0.589	94.20	64.71
Val	0.713	0.046	<0.001	0.623–0.804	0.374	55.07	82.35
(Leu + Val)/Tyr	0.735	0.046	<0.001	0.646–0.824	0.390	78.26	60.78
Pro	0.730	0.046	<0.001	0.639–0.820	0.376	76.81	60.78
Phe	0.612	0.051	0.036	0.512∼0.713	0.240	71.01	52.94
Ala	0.760	0.044	<0.001	0.674∼0.846	0.500	63.77	86.27
Gly	0.717	0.047	<0.001	0.625∼0.808	0.408	62.32	78.43

#### 3.4.3 Value of combined testing in differentiating the etiology of neonatal cholestasis

**Figure 3:**
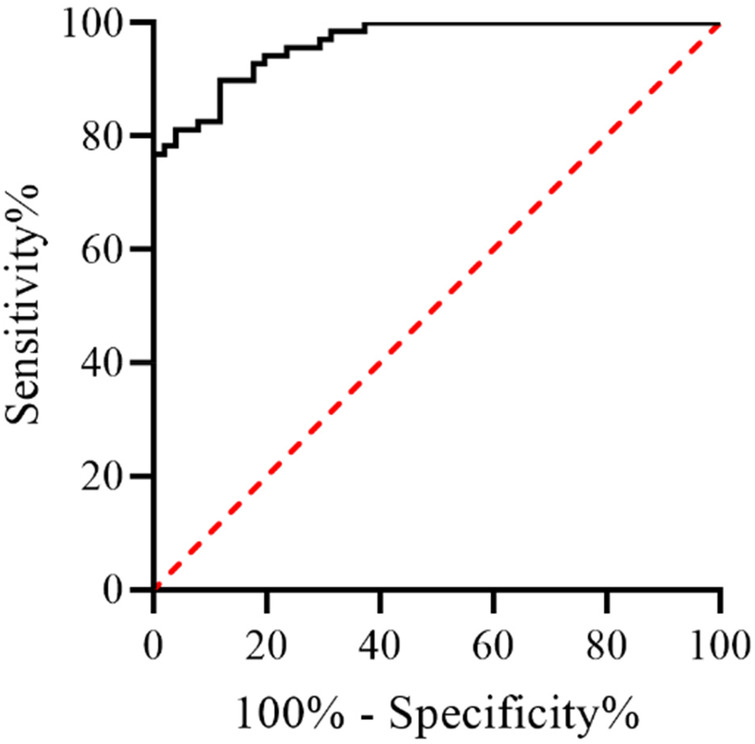
ROC curve of combined blood acylcarnitine and amino acid profiling for differentiating the etiology of neonatal cholestasis.

Based on the ROC curves of individual variables, markers showing better discriminatory ability (with AUC > 0.7 and *P* < 0.05) included C2, C12, Leu, Val, (Leu + Val)/Tyr, Pro, Ala, and Gly; these were selected to build an integrated logistic regression model for analysis of acylcarnitine and amino acid levels in this study. Using the predicted probabilities generated by the model as a combined diagnostic indicator, a ROC curve was constructed. These results showed that the integrated screening model achieved an AUC of 0.963 (95% CI: 0.937–0.990, *P* < 0.001), indicating strong diagnostic performance in differentiating the primary causes of neonatal cholestasis. The optimal cutoff value was determined using the Youden index (Youden index = sensitivity + specificity − 1) maximization principle. At the optimal cutoff value of 0.521 (Youden index = 0.781), the sensitivity and specificity of the combined model for differentiating biliary atresia were 89.86% and 86.24%, respectively. Detailed results are presented in Table 6 and Figure 3.

**Table 6:** Diagnostic value of combined blood acylcarnitine and amino acid profiling in differentiating the etiology of neonatal cholestasis.

Variable	AUC	SE	*P*	95% CI	Youden index	Sensitivity (%)	Specificity (%)
Combined	0.963	0.014	<0.001	0.937–0.990	0.781	89.86	86.24

### 3.5 Construction and validation of the combined detection model

#### 3.5.1 Logistic regression model parameters and model equation

Using biliary atresia as the dependent variable (coded as: biliary atresia group = 1, non-biliary atresia group = 0), and the eight candidate variables identified by univariate ROC analysis as independent variables, a binary logistic regression model was constructed. The regression coefficients (*β*), ORs, and 95% CIs of the model are summarized in Table 7. The formula for the combined detection model is:

**Table 7:** Parameters of the binary logistic regression model.

Variable	*β*	SE	Wald *χ*^2^	OR	95% CI	*P*
C2	1.247	0.342	13.291	3.478	1.780–6.795	<0.001
C12	0.026	0.301	0.007	1.026	0.569–1.850	0.932
Leu	−0.015	0.038	0.156	0.985	0.914–1.061	0.693
Val	0.033	0.041	0.648	1.034	0.953–1.121	0.421
(Leu + Val)/Tyr	0.210	0.067	9.824	1.234	1.082–1.408	0.002
Pro	−0.412	0.132	9.741	0.662	0.511–0.858	0.002
Ala	−0.098	0.029	11.418	0.907	0.857–0.960	0.001
Gly	0.024	0.011	4.760	1.024	1.002–1.046	0.029
Constant	−6.842	1.973	12.034	—	—	<0.001

#### 3.5.2 Model calibration and internal validation

##### 3.5.2.1 Model calibration assessment

The Hosmer–Lemeshow goodness-of-fit test was used to assess the calibration of the combined detection model. The test yielded a Hosmer–Lemeshow *χ*^2^value of 7.342 (*P* = 0.500), indicating no statistically significant difference between the predicted probabilities and the observed outcomes, suggesting that the model demonstrated good calibration.

##### 3.5.2.2 Model internal validation

Given the relatively limited sample size of this study (*n* = 180), internal validation was performed using the bootstrap resampling method to avoid model overfitting and to evaluate the stability and generalizability of the model. The corrected AUC was 0.949 (95% CI: 0.920–0.978), which decreased by only 0.014 compared with the original AUC of 0.963, indicating no significant overfitting and demonstrating good model stability and internal consistency. After bootstrap correction, the sensitivity and specificity of the combined detection model were 86.24% and 83.15%, respectively, slightly lower than those of the original model. The small fluctuations in each evaluation metric further confirmed the robustness of the model.

##### 3.5.2.3 Handling of missing data

All 180 neonates with neonatal cholestasis included in this study had complete clinical data and tandem mass spectrometry metabolomic results, with no missing data for blood acylcarnitine or amino acid profiles. A small number of missing values (all with missing rates < 5%) were identified for some biochemical indicators (e.g., GGT, ALP, AST, and ALT), which were imputed using the mean imputation method. Given the low missing rate, this method had a limited impact on the overall performance of the model.

## 4 Discussion

This study retrospectively reviewed the medical records and MS/MS metabolomic profiles of 180 neonates with cholestasis to assess the diagnostic value of combined acylcarnitine and amino acid profiling in differentiating biliary atresia from non-biliary conditions. The findings indicated some notable differences between the various causes in infants with jaundice of uncertain origin. The combined diagnostic model achieved an AUC of 0.963, considerably outperforming all individual metabolic markers. Therefore, the metabolic analysis method using MS/MS can be considered another tool for determining the cause of neonatal cholestasis.

Clinical biochemical indicators: The biliary atresia group showed significantly higher levels of the DBIL/TBIL ratio, GGT, and ALP compared with the non-biliary atresia group, which is consistent with previous studies (16, 17). An increased value of the DBIL/TBIL ratio indicates blockage of bile drainage as its essential biochemical manifestation. The difference reported by Güntur et al. was more prominent between groups than that of other indicators (*P* < 0.001). This agrees with the study by Güngör et al. (18), thus confirming that GT are useful indicators for diagnosing biliary obstruction. In addition, this study found that glucose concentrations in bile were significantly lower than in patients without cholangiopathies (*p* = 0.014), possibly due to greater liver damage, reduced glycogen reserves, and disrupted gluconeogenic metabolism in these patients. Alur et al. (19), in a study of cholestasis in very-low-birth-weight infants, found that hypoglycemia occurred at a rate of up to 54.5%, suggesting that hypoglycemia may represent an indispensable metabolic problem in infants with cholestasis.

With respect to the acylcarnitine profile, the concentrations of C2, C8, C12, and C18 were significantly higher in the biliary atresia group than in the non-biliary atresia group. However, differences among other components did not reach statistical significance. Acylcarnitines are products of abnormally elevated levels during impaired mitochondrial fatty acid oxidation (20, 21). The AUC-ROC for C2 and C12 were 0.767 and 0.742, respectively, indicating their lower discriminatory ability than others. In contrast to the research by Kido et al. (12), who reported that the combined Arg–Cit–Ile + Leu–Tyr–free carnitine-to-hexanoylcarnitine ratio exhibited some diagnostic value in NICCD, this study found no such effect under these circumstances. This discrepancy may be caused by different basic metabolic pathways. NICCD primarily involves disturbances in the urea cycle and fatty acid oxidation pathways, whereas the metabolic dysfunction of biliary atresia is likely to be more complex, including multisystem metabolic disorders secondary to cholangiopathy.

with respect to amino acid analysis, the contents of Leu, Val, Pro, Phe, Ala, and Gly and the (Leu + Val)/Tyr ratio were significantly higher in the biliary atresia group than in the non-biliary atresia group. The Fischer ratio, represented by the (Leu + Val)/Tyr ratio (often used for assessing liver function) is also included herein (22) and is significantly increased in this research context, thus supporting the hypothesis that the hepatic condition has worsened for those affected by biliary atresia. ROC analysis showed that Leu had the highest AUC of 0.805 and Ala had an AUC of 0.761, but Ala had lower sensitivity (86.27%) in terms of diagnostic validity. The levels of Cit, Orn, Arg, Met, and Tyr were not significantly different between the two groups. The citrate content in the non-biliary atresia patients (27.12 ± 10.53 μmol/L) remained within the normal range, indicating that fewer NICCD cases may be present at this time point.

The primary clinical value of this study lies in its combined detection method. The combined model, which included C2, C12, Leu, Val, the Fisher ratio, Pro, Ala, and Gly, achieved an AUC of 0.963 (95% CI: 0.937–0.990), which was more superior to that of any single metabolic component. A sensitivity of up to 89.86% was obtained. The particular recognizability reached at least one standard, specifically 86.24%. Du et al. (23) used non-targeted metabolomics to assess the metabolic signature of 90 children with biliary atresia and 48 infants with cholestatic jaundice, identifying 18 biomarkers with high diagnostic accuracy (AUC = 0.968). The above-mentioned experiments demonstrate the good applicability and accuracy of this method for diagnosing biliary atresia. The enhanced discriminatory ability of the combined detection method may come from two aspects: (1) acylcarnitines and amino acids reflect different biological functions; therefore, combining them provides more comprehensive metabolic information; (2) biliary atresia is a multifactorial disease associated with multiple metabolic disorders, which cannot be accurately reflected using a single biomarker alone.

This research has several limitations. First, it was a single-center retrospective design with a relatively small sample size, which may introduce selection bias and limit the generalizability of the results. Second, the etiological composition of the non-biliary atresia group is complex, including NICCD, infection-induced cholestasis, or idiopathic neonatal hepatitis. Moreover, due to the limited sample source and sample size, this study did not perform external validation and decision curve analysis. Therefore, the stability and generalizability of the combined model still require validation in independent cohorts. Finally, the combined model includes multiple metabolic indicators, and this study failed to establish a simplified scoring system, which may limit its convenience in clinical operation. Future studies could employ methods such as Least Absolute Shrinkage and Selection Operator (LASSO) regression to identify core indicators and develop a simplified scoring system, as well as conduct multicenter, large-sample prospective studies to further validate the diagnostic performance of the combined model.

In summary, adding acylcarnitine and amino acid profiling to the diagnostic criteria for infantile cholestasis is necessary. The proposed combined detection approach offers a novel system and method for the early detection and non-invasive risk classification of biliary atresia. Systematically applying tandem mass spectrometry metabolic analysis to neonatal jaundiced babies can facilitate prompt etiological diagnosis. In the future, there may be several multicenter, large-sample prospective studies investigating its diagnostic value. At the same time, it is necessary to develop a simple scoring system combining key metabolites to improve clinical utilization rates more efficiently.

## Data Availability

The raw data supporting the conclusions of this article will be made available by the authors, without undue reservation.
